# Genetic Diversity of 27 Y-STRs in Two Jordanian Subpopulations: Bedouins and Fellahin

**DOI:** 10.3390/genes17020194

**Published:** 2026-02-04

**Authors:** Almuthanna K. Alkaraki, Mohammad B. Alsliman, Mohammad M. Twait, Miguel A. Alfonso-Sánchez, Jose A. Peña

**Affiliations:** 1 Department of Biological Sciences, Faculty of Science, Yarmouk University, Irbid 21163, Jordan; alsalman411@gmail.com (M.B.A.); mohammadspecial021@gmail.com (M.M.T.); 2 Department of Genetics, Physical Anthropology and Animal Physiology, Faculty of Science and Technology, University of the Basque Country (UPV/EHU), Barrio Sarriena s/n, 48940 Leioa, Bizkaia, Spain; malfonsan@gmail.com (M.A.A.-S.); joseangel.pena@ehu.eus (J.A.P.)

**Keywords:** Y-STRs, Bedouins, Fellahin, population genetics, Jordan

## Abstract

Background/Objectives: The Bedouins (nomads) and the Fellahin (farmers) of Jordan represent two distinct subpopulations, characterized by unique lifestyles, settlement patterns, and linguistic features. This study aims to estimate the frequency of 27 Y-STRs in these two Jordanian subpopulations, along with various forensic parameters and paternal lineage comparisons with neighboring populations. Methods: Twenty-seven Y-STRs were typed in two major Jordanian subpopulations: Bedouin nomads (*n* = 101) and Fellahin farmers (*n* = 98). The forensic and paternal genetic lineage parameters and Y-haplogroup predictions were estimated. In addition, we conducted multidimensional scaling (MDS) and centroid analyses based on the Fst distance matrix to compare the sampled communities with neighboring populations from the MENA region, East Africa, Southeast Europe, and South Asia. Results: The Y-haplogroup predictions revealed differences in the predicted lineage composition based on the Y-STR profiles. The predicted J1a2a1a2 haplogroup predominated among the Bedouins (74.3%), whereas the Fellahin displayed a more heterogeneous profile, with notable frequencies of J1 (40%) and J2 (17.3%). Furthermore, the Fellahin exhibited remarkable genetic diversity and significant gene flow, providing plausible evidence of kinship with neighboring Levantine and Arabian groups. In contrast, the Bedouins showed consistently lower diversity across multiple loci, indicating long-term tribal isolation and, therefore, the potential effects of genetic drift. The MDS and centroid analyses positioned the Fellahin among the genetically interconnected Middle Eastern populations, while the Bedouins were clustered with the Arabian Peninsula populations. Conclusions: Overall, the contrasting genetic signatures of the two Jordanian subpopulations reflect their settlement patterns and sociocultural practices. In addition, the Y-STR dataset generated in this study enhances the Jordanian forensic database and to extends our understanding of paternal lineage structures in the West Asian/Levantine region.

## 1. Introduction

The Y chromosome constitutes a cornerstone of human evolutionary genetics; its paternal mode of inheritance and the lack of meiotic recombination in the male-specific region make it a powerful tool for forensic identification, tracing paternal ancestry, and reconstructing migration routes of past human populations [1,2]. Among its most informative features are short tandem repeats (Y-STRs), which provide insights into phylogenetic relationships and kinship patterns in both forensic and anthropological contexts [3,4,5]. The worldwide distribution of Y-STR variation has revealed evolutionary footprints of historical events, regional population expansions, nomadic mobility, and settlement processes [5,6,7,8,9].

Despite Jordan’s geographical position at the crossroads of Asia, Africa, and Europe [10], and the presence of diverse subpopulations and ethnic groups [11], genetic data from the Jordanian population remain limited.

Within Jordan, the Bedouins (nomads) and the Fellahin (farmers) represent the core of the original Trans-Jordan population, predating the major influxes of refugees during the 19th and 20th centuries as a result of political conflicts and regional wars [12,13,14,15]. These two socioculturally defined groups are distinguished by their lifestyles, settlement patterns, and linguistic features [16].

Bedouins (al-Badu) refers to the term Badawi, which means people who live in the Badia (desert) and belong to a qabila (tribe) as an inherent relationship [17]. A tribe embodies an assemblage of individuals forming a societal, cultural, economic, and political structure, typically inhabiting a singular geographic entity either of their volition or due to the influence of intertribal conflicts or external compulsion [18]. The Bedouins’ main activity is the farming of livestock like camels and sheep, and their basic diet depends on meat, fermented/dried dairy products, wheat, and imported dates [19]. On the other hand, the Fellahin’s livelihood depends mainly upon agricultural activity and small-scale animal husbandry including sheep, goats, and chickens. Generally, the Fellahin reside within villages in stone or mud houses, either with their immediate family or within an extended familial context (hamula/clan); the Fellahin‘s life revolves around this fundamental connection to the land [20]. Linguistically, the Fellahin speak a distinct Arabic dialect that is characterized by several phonetic features, such as using “Ani” instead of “Ana” for I am, and the addition of the phonetic sound of the letter “H” when addressing females. Moreover, they tend to pronounce the Arabic letter (ق, Gaf), as “g” in “big”, which is similar to Yemini and some Bedouins’ dialect, contrasting with the Druze (strongly articulated GAF), urban Levantine (pronounced as A), or Westbank Palestinians (pronounced as Kaf) [21]. In contrast, the Jordanian Bedouins’ dialect is somehow like that the Arabian Peninsula and retains many features of classical Arabic [22,23,24].

This study aims to estimate the frequency of 27 Y-STRs in two Jordanian subpopulations: Bedouins (nomads) and Fellahin (farmers). The findings are expected to provide new data for a Jordanian forensic Y-chromosome database and to refine the understanding of Y-STR and predicted Y haplogroups among both studied Jordanian subgroups, and in comparison with adjacent populations.

## 2. Materials and Methods

All scientific and experimental procedures followed the tenets of the Declaration of Helsinki and the Belmont Report for the protection of human subjects. The initial scientific proposal was carefully reviewed by the Institutional Review Board at Yarmouk University (IRB) to make sure it adhered to the standards’ ethical codes and the local cultural values (approval reference number: IRB/2023/13). Participation was voluntary, and all volunteers provided written informed consent before blood collection. The research team explained the study objectives and procedures in accessible language and shared the principal investigator’s contact information to safeguard the right to withdraw at any stage. The blood donors were randomly recruited from unrelated, healthy individuals aged 18–60 years. Sampling was conducted in the spring of 2023 (2 April to 29 May). The donors were classified according to their dialect, locality of residence, and family narrative history. The use of the terms Fellahin (farmers) and Bedouins (nomads) as descriptive categories aligns with Jordanian cultural norms and is regarded as a positive marker of local heritage, raising no ethical concerns regarding discrimination. The Fellahin samples were collected from subjects residing in the northern Jordanian governorates (Irbid, Jerash, and Ajloun), whereas the Bedouin samples were collected from the three administratively defined (virtual) Bedouin regions (northern, central, and southern). Three subjects with ambiguous backgrounds were excluded, which accounted for 0.014% of the initial recruitment pool. The exclusion criteria included individuals of non-Trans-Jordanian paternal ancestry who did not self-identify as Arab and therefore could not be assigned to either studied subgroup. In total, 3 mL of venous blood was collected in EDTA tubes from 199 Jordanian males, comprising 98 Fellahin and 101 Bedouins. Donor privacy was protected by coding all the samples, and access to the genomic and demographic data was restricted to the principal investigator.

The genomic DNA was extracted from the blood samples with a Monarch^®^ Genomic DNA Purification Kit (New England Biolabs, Ipswich, MA, USA), following the manufacturer’s instructions. The DNA quantity and quality was measured using a commercial “Investigator Quantiplex Pro Kit” (Qiagen, Venlo, The Netherlands) according to the manufacturer recommendations. Twenty-seven Y-STR loci were amplified using a Yfiler™ Plus PCR Amplification Kit (Applied Biosystems, Waltham, MA, USA). Capillary electrophoresis was performed on a 3500XL Genetic Analyzer, and alleles were assigned with GeneMapper™ ID-X Software v1.6 (Thermo Fisher Scientific, Waltham, MA, USA), based on the allelic ladder provided by the kit. After experimental optimization, a total of 10 random samples were genotyped twice to ensure quality assurance along with performing standardization of known samples as a control.

Allele frequencies for each locus were calculated by using IBM SPSS Statistics v25. Gene diversity (formula: D = (n/n − 1) × (1 − ∑pi2)) was computed manually for each locus. NEVGEN Haplogroup Predictor (Y-DNA Haplogroup Predictor—NEVGEN.ORG) was used to predict Y haplogroups. Genetic distances (Fst) and centroid analyses were performed with GenoCline software package [25]. Multidimensional scaling (MDS) and neighbor joining (NJ) analyses were conducted using PAST program [26]. In order to establish population clusters, series of iterations were performed with Structure [27] for different values of K. Results were analyzed with structure Harvester [28] to determine most plausible number of clusters.

## 3. Results

### 3.1. Allele Frequencies and Genetic Diversity

The data on 27 Y-STR loci were obtained from 199 Jordanian males (Supplementary Materials Table S1). The allele frequencies and genetic diversity (GD) indices were calculated for the single-copy markers (23 Y-STRs) in the Fellahin (Supplementary Materials Table S2) and Bedouin (Supplementary Materials Table S3) subpopulations. The GD values ranged from 0.114 (DYS392) to 0.833 (DYS458) in the Bedouins, and from 0.305 (DYS392) to 0.880 (DYS458) in the Fellahin. The number of alleles per locus varied from 3 (DYS389I) in both subpopulations to 14 (DYS449) in the Fellahin and 11 (DYS458) in the Bedouins. Both the Fellahin and Bedouins showed high GD values (>0.750) at DYS576, DYS627, DYS481, and DYS518. Conversely, the Bedouins exhibited very low GD (<0.300) at DYS392, DYS437, and DYS533, while the Fellahin showed relatively low GD (0.300–0.500) at DYS437, DYS389I, and YGATAH4. Overall, the Fellahin displayed greater GD in 19 STRs, whereas the Bedouins showed higher diversity in 4 STRs.

An analysis of multi-copy loci (Supplementary Materials Table S4) revealed extensive allelic variation in both groups. At the DYS385 locus, 33 allelic combinations were identified in the Fellahin compared to 24 in the Bedouins. At DYS387S1, the Fellahin exhibited 23 allelic patterns, while the Bedouins showed 13. Both loci (DYS385 and DYS387S1) demonstrated high GD in the Fellahin (>0.850), highlighting their strong power of discrimination among unrelated male lineages. Similarly, the Bedouins displayed high GD at DYS385 (0.866), whereas the GD at DYS387S1 was comparatively lower (0.806).

The heterogeneity between the two subpopulations in Jordan (Bedouin and Fellahin) was analyzed. It was found that they were significantly different (Mann–Whitney U-test: z = 2.91; *p* < 0.01).

### 3.2. Y Haplogroups

Twenty-three of the twenty-seven markers were used to predict the Y haplogroups with the NEVGEN Y-DNA Haplogroup Predictor online tool. The analysis identified 17 distinct haplogroups belonging to eight macro-haplogroups in the Fellahin subpopulation, compared to 15 distinct haplogroups within eight macro-haplogroups in the Bedouin subpopulation (Table 1). The predominant haplogroup in the Bedouins was J1a2a1a2, accounting for 74.3% of sampled individuals. The same haplogroup was also detected in the Fellahin, but at a lower frequency (40%). Haplogroup J2 was present in 17.3% of Fellahin compared to only 3.9% of Bedouins. Haplogroup E occurred in 17% of Fellahin and 4.9% of Bedouins. Haplogroup A showed comparable frequencies in both groups (3%). All other haplogroups were observed at frequencies below 5% in both populations. Additionally, the predictor classified one sample from each subpopulation as belonging to an unsupported subclade.

**Figure 1:**
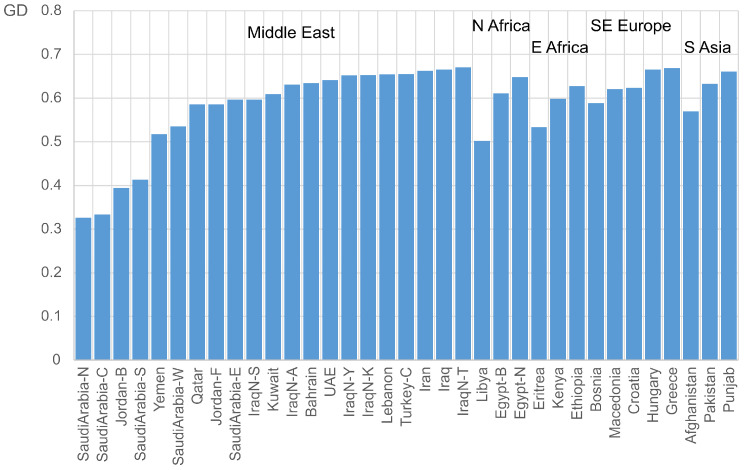
Genetic diversity observed in a series of populations from the Middle East, North Africa, East Africa, Southeast Europe, and South Asia for a group of STRs on the Y chromosome. Population labels: N (North), E (East), S (South), SE (Southeast), W (West), Jordan-B (Jordanian Bedouins), Jordan-F (Jordanian Fellahin), IraqN-S (Northern Iraqi Syriacs), IraqN-A (Northern Iraqi Arabs), IraqN-Y (Northern Iraqi Yazidis), IraqN-K (Northern Iraqi Kurds), IraqN-T (Northern Iraqi Turkmens), Turkey-C (Central Turkey), Egypt-B (Egypt Berbers), and Egypt-N (Northern Egyptians).

A database was compiled containing the allele and haplogroup frequencies from populations in nearby regions, including the Middle East [29,30,31,32,33,34,35,36,37,38,39], North Africa [40], East Africa [41], Southeast Europe [36], and South Asia [42,43,44]. A comparative analysis with these populations indicated that the genetic diversity of the Jordanian Bedouins (Jordan-B) is relatively low (Figure 1).

**Figure 2:**
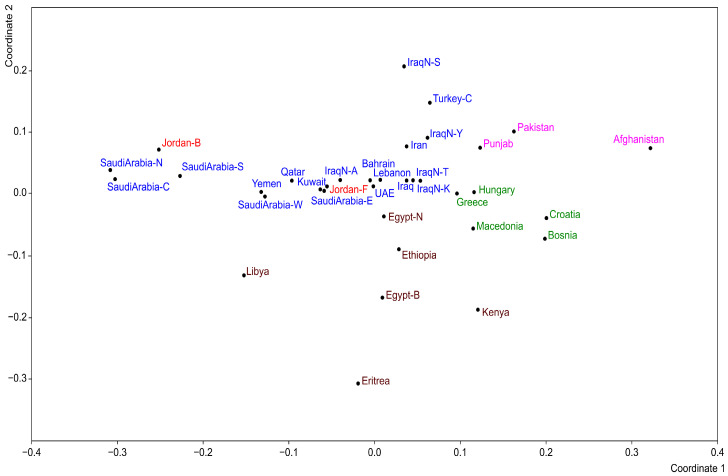
MDS on FST distance matrix based on allele frequencies. Populations of South Asia are identified in fuchsia, those of Europe in green, those of North and East Africa in brown, those of Middle East in blue, and those of Jordan in red.

Indeed, it ranks among the lowest values recorded, comparable to those observed in several populations across Saudi Arabia (north, center, and south). Conversely, the value observed in the Fellahin population (Jordan-F) is higher and approximates the average of the populations considered in this study. The MDS plot derived from the Fst distances based on the allele frequencies is shown in Figure 2. The populations from South Asia (fuchsia), Europe (green), and North and East Africa (brown) are primarily distributed along the positive segment of Axis 1. Within this distribution, the South Asian populations are positioned at the top of the graph, the European populations occupy a central position, and the African populations are located at the bottom. Afghanistan, Eritrea, and Libya are the most distant from these clusters. Afghanistan lies at the positive end of Axis 1, Eritrea at the negative end of Axis 2, and Libya is relatively close to the populations of the Arabian Peninsula. The Berbers of Egypt (Egypt-B) are separated from the Arab populations and positioned among those of East Africa. Most Middle Eastern populations cluster around the centroid, including the Fellahin of Jordan (red). The most distant populations within this group are the Turks (Turkey-C) and the Syriacs of Iraq (IraqN-S), located at the positive end of Axis 2. At the negative end of Axis 1 are three Saudi Arabian populations (north, center, and south) together with the Bedouins of Jordan. This cluster of populations exhibits the lowest genetic diversity (Figure 1).

**Figure 3:**
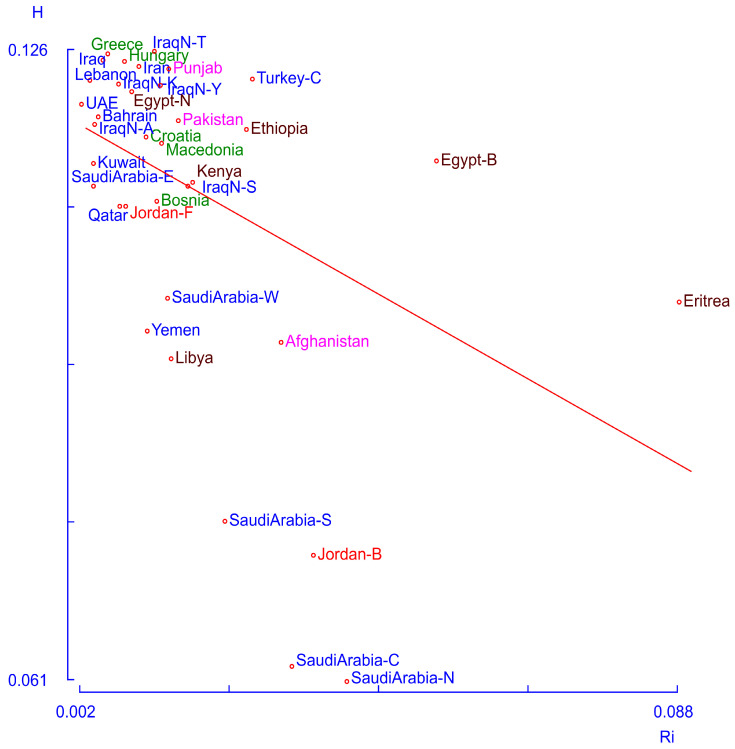
Centroid analysis of a group of populations from South Asia, the Middle East, Europe, and northeast Africa. Populations of South Asia are identified in fuchsia, those of Europe in green, those of North and East Africa in brown, those of Middle East in blue, and those of Jordan in red. The line represents the expected relationship between heterozygosity and Ri.

The centroid analysis (Figure 3) reveals a large cluster of populations with similar features, characterized by high heterozygosity (H) and low heterogeneity (Ri) values. These findings suggest a substantial level of gene flow among them, and the Fellahin population of Jordan is included in this cluster. The Eritreans and Egyptian Berbers exhibit both high heterogeneity and heterozygosity, likely reflecting gene flow from populations not represented in the database. In contrast, three Saudi Arabian populations (north, center, and south), together with the Bedouins of Jordan, display very low heterozygosity and average heterogeneity values, indicating a high degree of population isolation.

**Figure 4:**
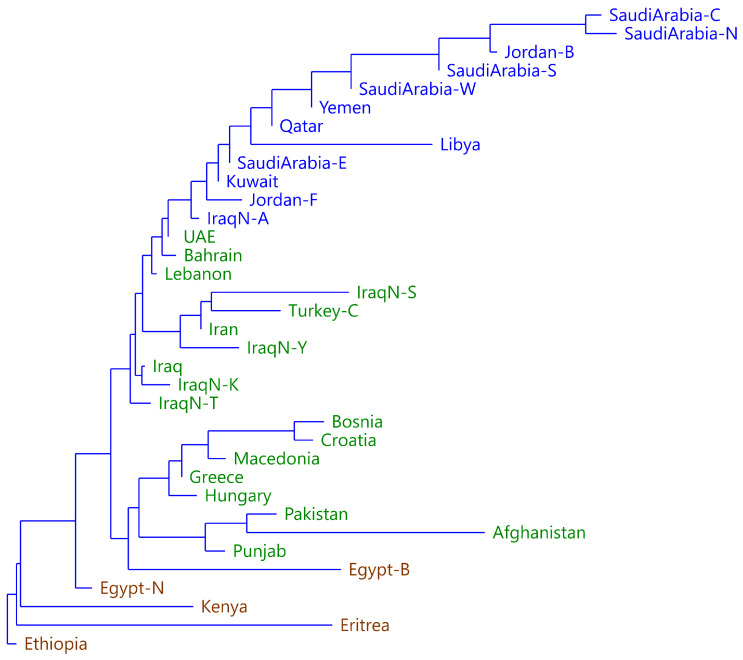
NJ on FST distance matrix based on allele frequencies. Populations are colored according to the predominant component in Structure for K = 3. Group 1 is colored green, group 2 blue, and group 3 brown.

A series of analyses were performed using Structure with different values of K and several repetitions. The results were analyzed using Structure Harvester, which found that the best fit was obtained for K = 3. An NJ tree was also obtained from the FST distance matrix using Past (Figure 4). The population labels are colored according to the most important Structure component in each population. The groupings are distributed consistently. One group (brown) includes populations from North and East Africa, with the exception of Libya, which has an Arab origin. A second group (green) includes European populations, populations from South Asia, and some populations from the Middle East. The third group (blue) finally includes most of the populations of the Arabian Peninsula and Libya.

**Figure 5:**
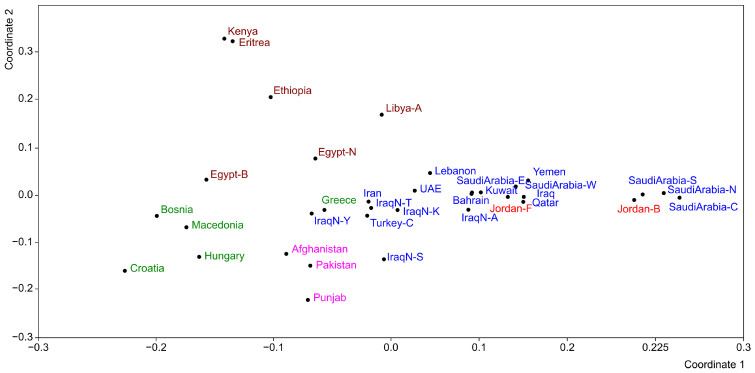
MDS on FST distance matrix based on haplogroup frequencies. Populations of South Asia are identified in fuchsia, those of Europe in green, those of North and East Africa in brown, those of Middle East in blue, and those of Jordan in red.

An MDS analysis was also performed using the haplogroup frequencies for the same set of populations (Figure 5). The results closely parallel those emerging from the allele frequencies. Axis 1 distributes the South Asian populations (fuchsia), European populations (green), and North and East African populations (brown) toward one end, corresponding in this case to negative values. In this analysis, however, Afghanistan does not appear significantly differentiated from Punjab and Pakistan. The Fellahin of Jordan again occupy an intermediate position, clustering with a large group of Middle Eastern populations. Finally, the northern, central, and southern Saudi Arabian populations, together with the Bedouins of Jordan, are located at the opposite end of Axis 1, once again standing out for their distinct genetic profile.

## 4. Discussion

The present study provides a comprehensive overview of Y-STR diversity in the Fellahin and Bedouin subpopulations of Jordan. Historically, the studied subgroups are considered as the main native population of Trans-Jordan before the multi-waves of refuges, such as Arab (Palestinians, Iraqi, and Syrians) and non-Arab refuges (Chechen, Circassian, and Armenian).

The obtained results clearly demonstrate a difference between the Bedouins and Fellahin in the predicted Y-STR haplogroups. These differences reflect a contrasting paternal demographic history shaped by the settlement patterns, social organization, and acceptance of others within agricultural communities vs. the restricted tribal organization associated with Bedouin culture.

The predicted predominant haplogroup J1 observed in the Jordanian Bedouins supports a paternal lineage continuity with the Arabian Peninsula, particularly the J1-P58 branch, which is associated with Semitic-speaking pastoral tribes and underwent major expansion during the Holocene [45]. In addition, higher frequency of such predicted haplogroups has been reported in Saudi Arabia, Yemen, and the Gulf area, often linked with a tribal founder effect [34,35]. Compared to the Bedouins, the predicted haplogroups in the Fellahin show a more balanced composition (J1, J2, and E), indicating an acceptable gene flow within the Fellahin subpopulation. The J2 haplogroup is associated with Neolithic agricultural expansion from the Fertile Crescent, while the reported haplogroup E reflects the gene flow between north/east Africa and the Levant area, likely facilitated by climate-driven migration or trade routes [40,46]. Other minor haplogroups reported in the Fellahin reflect admixture and ongoing gene flow.

The lower GD observed among the Bedouin subpopulation across many loci is consistent with tribal structures and paternal lineage-based stratifications. Tribal Bedouins favor the expansion of a limited number of male founders and reduce the male ancestral size, resulting in amplified genetic drift. A similar reduction in Y-chromosome diversity can be noticed in the Arabian Peninsula populations, where tribal identity and consanguineous marriages shaped the tribal structure to depend on the paternal lineage [34,35]. Moreover, low Y-STRs diversity is always associated with closed/isolated populations with strong genealogical continuity traced through the male lineage [7].

In contrast, the observed higher diversity in the Fellahin subgroup reflects a more heterogeneous paternal composition and a more diverse gene pool. Historically, agricultural communities engaged in trade, urban interactions, and intercommunity marriages, all of which promote male-mediated gene flow. Comparable Y-STR diversity patterns have been reported in Levantine communities with long-term settlement, including Syrian, Lebanese, and Mesopotamian groups [32,33,47,48].

The two studied subpopulations were analyzed within a broad geographical framework, considering comparative data from Asia, Europe, and Africa to explore potential genetic affinities. The multidimensional scaling based on both alleles and predicted haplogroups groups the Fellahin with other Levantine populations and broader Middle Eastern populations, while the Bedouins are aligned more closely with Arabian Peninsula groups. Thus, the male lineages of the Fellahin exhibit a greater genetic diversity level within the ranks of the analyzed population set. This pattern points to a complex demographic history involving admixture events and long-term gene flow with neighboring communities. Their agricultural tradition has kept them rooted to the land and sharing in the demographic processes that have unfolded in the region over time. Jordan’s geographical position at the intersection of Africa, Asia, and Europe has made the region a historical arena for numerous transformative events, including major migratory movements since the out-of-Africa dispersal. It is further acknowledged as one of the principal centers of origin of agriculture and animal husbandry, practices that subsequently diffused from the Middle East into Europe and, likely, into North and East Africa and southwest Asia. The Middle East was among the few regions where cattle domestication occurred. Nevertheless, both agriculture and pastoralism underwent a substantial decline as a result of widespread aridification. The conclusion of the African Humid Period precipitated the rapid desertification of the Sahara and the Arabian Peninsula [49,50] with profound consequences, including the collapse of the Akkadian Empire [51] and the Old Kingdom in Egypt [52] around 4300 years ago.

It has been argued that the aridification of the Arabian Peninsula may have precipitated a subsistence crisis, which was alleviated through the domestication of the camel [53]. The demographic pressures arising from the depletion of agricultural and pastoral resources, combined with the low population densities inherent to resource-poor environments, represent two plausible drivers of the pronounced genetic drift observed in populations that persisted in desert contexts, such as the Bedouins. The imprint of this drift is evident in the markedly low genetic diversity and high genetic heterogeneity relative to neighboring populations, as demonstrated by the multidimensional scaling (MDS) analyses of both allele and haplotype frequencies.

From a forensic point of view, the higher haplotype diversity among the Fellahin increases the discriminatory power; meanwhile, the reduced diversity among the Bedouins may increase haplotype sharing among different individuals belonging to the same tribal background. This pattern is recognized in forensic interpretations of endogamous populations.

## 5. Conclusions

This study generated a comprehensive dataset of 27 Y-chromosomal markers in two Jordanian subpopulations, the Bedouins and the Fellahin. The genetic relationships observed between both groups and with neighboring populations are congruent with the geographic, linguistic, and cultural frameworks that have historically shaped the region. Notably, the Bedouin haplotypes reflect reduced Y-STR haplotype diversity consistent with limited paternal lineage variability, whereas admixture, gene flow, and demographic complexity are reflected in the Fellahin subpopulation. The Y-STR profiles obtained for both subpopulations not only enhance the resolving power of the Jordanian forensic database, but also contribute to the reconstruction of paternal lineages, the refinement of our understanding of population structure, and the enrichment of the evolutionary narrative of Middle Eastern and Levantine groups.

## Figures and Tables

**Table 1:** Frequencies of predicted haplogroups in Jordanian Fellahin and Bedouin populations.

Fellahin		Bedouins	
Predicted Haplogroup	Frequency	Predicted Haplogroup	Frequency
A0a	0.010204	A1b1b2b	0.029703
A1b1b2b	0.020408	E1a	0.009901
E1a	0.010204	E1b1b	0.039604
E1b1b	0.173469	G2a	0.019802
G2a1	0.010204	G2a2b1	0.009901
G2a2b1	0.030612	G2b	0.019802
H1a1a	0.010204	H1a1a	0.009901
I2a2a	0.010204	J1a	0.009901
J1a	0.010204	J1a2a1a2	0.742574
J1a2a1a2	0.408163	J2a1	0.039604
J1b	0.010204	L1a	0.009901
J2a1	0.142857	R1a	0.009901
J2b2a	0.020408	R1b	0.009901
J2b2b	0.010204	R2	0.009901
R1a	0.05102	T	0.019802
R1b	0.020408		
T	0.040816		

## Data Availability

The original contributions presented in this study are included in the article/Supplementary Materials. Further inquiries can be directed to the corresponding author.

## Supplementary Materials

The following supporting information can be downloaded at: https://www.mdpi.com/article/10.3390/genes17020194/s1, Table S1: Raw data for the 27 Y-STRs in both Jordanian Bedouins and Fellahin subpopulations. Table S2: Allele frequencies and genetic diversity (GD) for single-copy marker in Fellahin Jordanian population. Table S3: Allele frequencies and genetic diversity (GD) for single-copy marker in Bedouin Jordanian population. Table S4: Allele frequencies and genetic diversity (GD) for Multi-copy marker in Fellahin and Bedouin Jordanian population.
